# First Report of *Wenzhou sobemo-like virus 4* in *Aedes albopictus* (Diptera: Culicidae) in Latin America

**DOI:** 10.3390/v14112341

**Published:** 2022-10-25

**Authors:** Pâmela S. Andrade, Ian N. Valença, Marta R. S. Heinisch, Esmenia C. Rocha, Lícia N. Fernandes, Nuno R. Faria, Ester C. Sabino, Tamara N. Lima-Camara

**Affiliations:** 1 Department of Epidemiology, Faculdade de Saúde Pública, Universidade de São Paulo, São Paulo 01246-904, Brazil; 2 Institute of Tropical Medicine, Faculdade de Medicina, Universidade de São Paulo, São Paulo 05403-000, Brazil; 3 Department of Infectious and Parasitic Diseases, Faculdade de Medicina, Universidade de São Paulo, São Paulo 01246-903, Brazil; 4 Medical Research Laboratory 49, Institute of Tropical Medicine, Faculdade de Medicina, Universidade de São Paulo, São Paulo 01246-903, Brazil; 5 MRC Centre for Global Infectious Disease Analysis, Jameel Institute, Department of Infectious Disease Epidemiology, School of Public Health, Imperial College London, London W2 1PG, UK

**Keywords:** insect-specific viruses, *Aedes albopictus*, vectors, metagenomics

## Abstract

Insect-specific viruses (ISVs) are viruses that replicate exclusively in arthropod cells. Many ISVs have been studied in mosquitoes as many of them act as vectors for human etiological agents, such as arboviruses. *Aedes (Stegomyia) albopictus* is an important potential vector of several arboviruses in Brazil, such as dengue (DENV), Zika (ZIKV) and chikungunya (CHIKV). The development of next-generation sequencing metagenomics has enabled the discovery and characterization of new ISVs. *Ae. albopictus* eggs were collected using oviposition traps placed in two urban parks in the city of São Paulo, Brazil. The *Aedes albopictus* females were divided into pools and the genetic material was extracted and processed for sequencing by metagenomics. Complete genomes of ISV *Wenzhou sobemo-like virus 4 (WSLV4)* were obtained in three of the four pools tested. This is the first detection of ISV *WSLV4* in *Ae. albopictus* females in Latin America. Further studies on ISVs in *Ae. albopictus* are needed to better understand the role of this species in the dynamics of arbovirus transmission in the Americas.

## 1. Introduction

Insect-specific viruses (ISVs) are viruses that replicate exclusively in arthropod cells and cannot infect vertebrates [1]. In mosquitoes, ISVs are transmitted vertically, being found in eggs, larvae, and adults; and horizontally, during mating [2]. Because ISVs are strongly associated with and adapted to mosquitoes, they form a unique virome, usually identified as an RNA (ribonucleic acid) virus [3]. The development of next-generation sequencing metagenomics has enabled the discovery and characterization of new ISVs, especially in mosquitoes that act as vectors for human pathogens. Public health applications of ISVs are currently being investigated. Some examples include the impact of ISVs on the ability of mosquitoes to infect and transmit certain arboviruses and on the longevity or fecundity of these mosquitoes [2]. Another potential application relates to creating arbovirus vaccines, using an ISV as a platform and recombining it with arboviruses of the same genus [4].

The *Aedes (Stegomyia) albopictus* (Skuse) mosquito is found in tropical, subtropical, and temperate areas around the world, where it can act as a major vector of dengue virus (DENV), chikungunya virus (CHIKV), and Zika virus (ZIKV) [5,6]. In Brazil, *Ae. albopictus* is considered a potential vector of these arboviruses, although natural vertical transmission of DENV and ZIKV has already been reported in this species [7,8]. The rapid spread of *Ae. albopictus* in Brazil is remarkable. It was first detected in 1986 and is currently present in all regions of Brazil. In addition to its confirmed vector competence for DENV, CHIKV, ZIKV, and yellow fever virus (YFV), *Ae. albopictus* has great ecological significance as it has already been found in wild, rural, and urbanized areas, and females take blood meals not only on humans but also on other vertebrates, making this species a possible bridge for viruses circulating from wild to urban environments, such as YFV [8].

The ISVs in *Ae. albopictus* have been studied in some parts of the world [9] and this species seems to have a stable central virome as some ISVs have been found in all life stages of this mosquito [9,10]. WSLV4 was first found in mosquitoes in China in 2013 but without species description [11].

Although *Ae. albopictus* is considered a potential vector of arboviruses in Brazil, it is important to know more about the ISVs harbored by this species to better understand the role it may play in the dynamics of arbovirus circulation in nature.

In this communication, we report for the first time the occurrence of the ISV *WSLV4* in the population of *Ae. albopictus* from an urban area in Brazil.

## 2. Materials and Methods

### 2.1. Sample Collection

**Figure 1:**
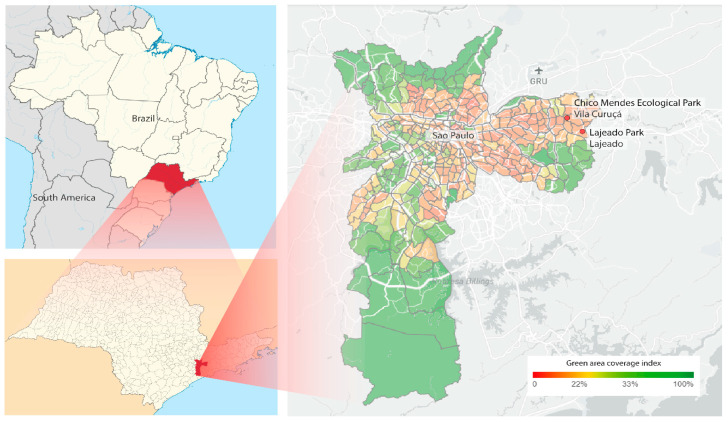
Left above: Map of Brazil with the state of São Paulo highlighted; Below: map of the state of São Paulo with the city of São Paulo highlighted. Right: Location of the Ecológico Chico Mendes and Lajeado-Izaura Pereira de Souza Franzolin urban parks (in the red circles), in the city of São Paulo, São Paulo state, Brazil.

*Ae. albopictus* eggs were collected from oviposition traps in two urban parks: Chico Mendes Ecological Park, in Vila Curuçá (23°30′25.7″ S 46°25′39.2″ W), and Lajeado-Izaura Pereira de Souza Franzolin Park, in Lajeado (23°32′18.3″ S 46°24′18.6″ W), both in the eastern part of the city of São Paulo (Figure 1). Collections were made in all seasons for three consecutive weeks between October 2018 and September 2019. The collected eggs were taken to the Public Health Laboratory of the Faculty of Public Health of the University of São Paulo, where they were hatched, and the species was identified during the larval and pupal stages. Subsequently, the females of *Ae. albopictus* were randomly selected and divided into pools of 10 individuals each, taking into account the season, year, and place of capture. Pools were stored in a freezer at −80 °C for virome evaluation.

### 2.2. Sample Processing

All pools were macerated in PBS buffer and centrifuged at 270 RCF (6000 rpm) for 15 min at 4 °C. Two hundred microliters of the supernatant were used for extraction of nucleic acids with the semi-automated DNA and RNA Extractor and Purifier apparatus EXTRACTA 96 (Loccus), with the fast DNA and viral RNA kit MXVA-P096 FAST (Loccus, Cotia, São Paulo, Brazil). The final elution of 50 µL of the final product was stored in a freezer at −80 °C.

We used the metagenomics technique SMART (switching mechanism at the 5′ end of RNA template)-9n [12] in samples from mosquito pools, focusing on RNA. Forty-four microliters of the extracted genetic material were treated with Turbo-DNase (Thermo Fisher Scientific, Waltham, MA, USA) at 37 °C for 30 min to remove DNA. RNA was then purified and concentrated using the Zymo RNA clean-up and concentrator-5 kit (Zymo Research, Irvine, CA, USA) according to the manufacturer’s instructions, with the final elution in 11 µL. For cDNA synthesis, we used 10 µL of each sample with 1 µL of deoxyribonucleotide triphosphate (dNTP) mixture (10 mM) (Cat. No. N0447L, New England BioLabs, Ipswich, MA, USA) and 1 µL of 9N NEB-RT labeled primer (2 µM) (AAGCA GTGGT ATCAA CGCAG AGTAC NNNNN NNNN), where they were incubated at 65 °C for 5 min and then cooled on ice. A mixture containing 4 µL SuperScript IV buffer, 1 µL DTT (0.1 M), 1 µL RNase OUT, 1 µL SSP primer (2 μM) (GCTAA TCATT GCAAG CAGTG GTATC AACGC AGAGT ACATrGrGrG), and 1 µL SuperScript IV (Cat. No. 18091200, Thermo Fisher Scientific, Waltham, MA, USA) was then prepared.

Samples were incubated at 42 °C for 90 min and at 70 °C for 10 min. The PCR reaction was performed with 5 µL Q5 reaction buffer (NEB, Ipswich, MA, USA), 0.5 µL 10 mM dNTP, 1 µL NEB PCR primer (20 μM) (AAGCA GTGGT ATCAAC GCAGA GT), 15.75 µL nuclease-free water, 0.25 µL Q5 DNA polymerase (NEB, Ipswich, MA, USA) and 2.5 µL sample. The cycle for total amplification corresponded to the following parameters: 1 cycle at 98 °C for 45 s, followed by 30 cycles of 98 °C for 15 s, 62 °C for 15 s, and 65 °C for 5 min and then terminated at 65 °C for 10 min. The amplified products were purified using 1× AMPure XP beads (Cat. No. A63881, Beckman Coulter, Brea, CA, USA) and quantified with a Qubit dsDNA High Sensitivity fluorometric assay (Cat. No. Q32854, Life Technologies, Carlsbad, CA, USA) on the Qubit 3.0 instrument (Life Technologies, Carlsbad, CA, USA), both according to manufacturer’s instructions.

### 2.3. Nanopore Library Preparation and Sequencing

Libraries were prepared using 50 ng of each amplified sample. The barcode identification of each sample was performed using the EXP-NBD104 (1-12), EXP-NBD114 (13-24), and Native Barcoding (ONT, Oxford, UK) kits. An SQK-LSK109 kit was used to prepare the library. Fifty nanograms of the final library was applied to the flow cell FLO-MIN106 inserted into the MinION (ONT, Oxford, UK) instrument and sequenced using the MinKNOW program, which ran for 12–24 h.

### 2.4. Bioinformatic Workflow

The FAST5 files (raw files) generated during sequencing were converted to FASTQ (base-calling files) using Guppy software version 2.2.7 GPU basecaller (ONT). They were then separated by barcode using Guppy Barcoder version 6.0.7 (ONT). NanoStat software version 1.1.2 was used to calculate the number of reads of each sample, the number of raw reads, and the minimum length of the contig to cover 50% of the genome. Then, each sample was taxonomically classified using the MiniKraken2_v1_8GB Kraken 2 database. The identified *WSLV4* was mapped against the reference genome (GenBank accession NC_033138.1) using Minimap2 version 2.28.0 and converted to a BAM file using SAMtools version 1.16. Genome mapping was performed using Tablet version 1.19.05.28. For consensus generation, we used SAMtoolsmpileup, which produces “pileup” textual format from an alignment, and Seqtk Seq to convert the fastq file into fasta. The similarity was checked using BLASTX version 2.13.0+ [13].

### 2.5. Phylogenetic Analysis

Multiple sequence alignment was done with MAFFT version 7.450 [14] using 3 complete genomes of *WSLV4* generated in this study and 45 closely related viral sequences retrieved from NCBI Blastx using all hits reported. A maximum likelihood tree was reconstructed using IQ-TREE version 1.6.12 [15] under the GTR+R+R8 nucleotide substitution model chosen as the best fitting model according to the Bayesian information criteria through ModelFinder. Bootstrap support was done with 1000 replicates. Internal nodes with >75% statistical support were assigned to the tree.

## 3. Results

The viromes of four pools of *Ae. albopictus* females from two urban parks in the city of São Paulo (Table 1) were analyzed using metagenomics. Classification of data generated in the MiniKraken2_v1_8GB Kraken 2 database identified reads that showed greater similarity to *WSLV4* in three pools (coverage from 99.73% to 100%), two from females from Chico Mendes Ecological Park and one from Lajeado. One pool of females from Chico Mendes Ecological Park had no reads similar to this virus (Table 1).

**Figure 2:**
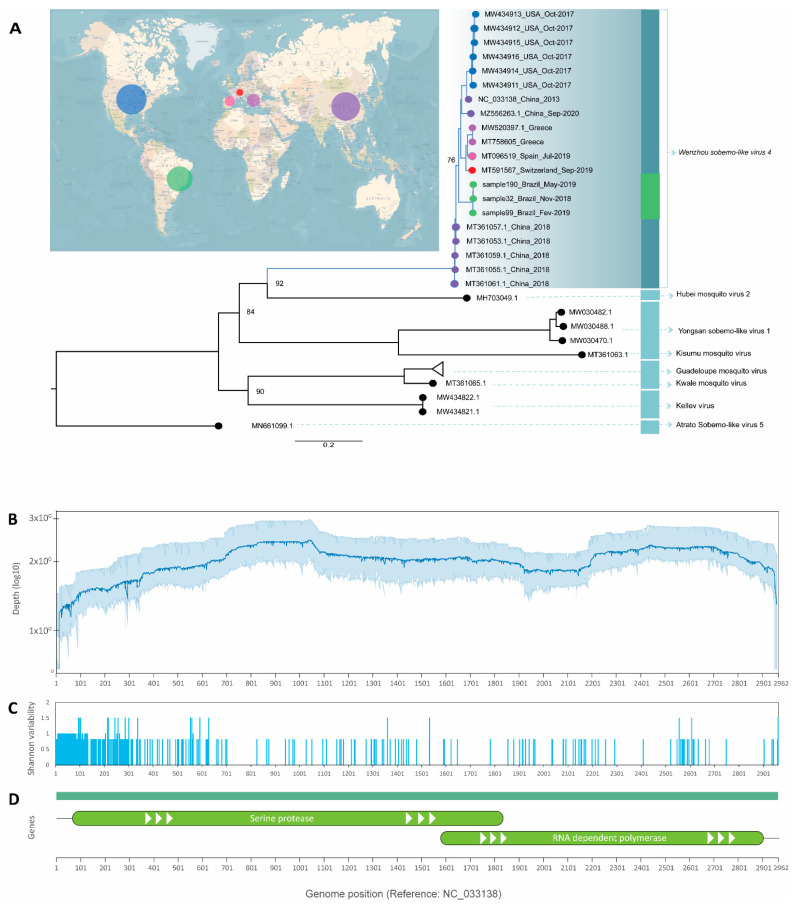
(**A**) Maximum-likelihood tree based on complete genomes of all closely related hits from Blastx to *WSLV4* genomes generated in this study. Circles on the map are approximately scaled to the number of genomes of *WSLV4* represented on the tree. Scale is in substitutions per site. (**B**) Average depth for the three *WSLV4* genomes and the respective 95% confidence intervals. (**C**) Shannon entropy values across the genomes showing regions of nucleotide dissimilarity to the reference. (**D**) Genes known to be expressed by *WSLV4* and their relative position on the genome.

The phylogenetic analysis revealed sequences previously submitted to the NCBI with tentatively different names. *Nea chili luteo-like virus*, *Sichuan mosquito sobemo-like virus* and *Guangzhou sobemo-like virus* (GenBank accession number MW520397.1, MZ556263.1, and MT361055.1, respectively) clustered together in a monophyletic branch with *WSLV4*, with high statistical support, strongly suggesting they are likely to belong to the same virus species (Figure 2A–C).

After mapping and visualization against the reference genome of *WSLV4* (GenBank accession number NC_033138.1), consensus sequences were generated. *WSLV4* belongs to the genus *unclassified RNA viruses ShiM-2016*. It has a simple complete genome with only 2961 bp translating two proteins (Figure 2D). When protein similarity was checked using BLASTX [13], samples 32, 99, and 190 were found to be 89.24%, 94.34%, and 97.79% similar to hypothetical protein 1 (GenBank accession number YP_009337375), respectively. As for hypothetical protein 2 (GenBank accession number YP_009337376), samples 32, 99, and 190 exhibited 99.09%, 99.32%, and 99.55% similarity, respectively. To the best of our knowledge, this virus has never been reported before in mosquito populations in Latin America.

## 4. Discussion

Mosquitoes can act as vectors for several human pathogens, such as arboviruses. *Ae. albopictus* is a vector of DENV, ZIKV, and CHIKV in some countries [5], but in Brazil it is considered a potential vector of these arboviruses [8]. Recent studies in various geographic regions have been conducted to report ISVs in *Ae. albopictus* [9]. However, there is still little information on ISVs in *Ae. albopictus*, possibly due to its lesser importance as a vector compared to *Aedes aegypti* [9,10].

In this study, ISV *WSLV4* was detected for the first time in Latin America in pools of female *Ae. albopictus* through next-generation sequencing technology using a metagenomics methodology. *WSLV4* is an ISV that is tentatively nonpathogenic to humans. There have been no reports of infection by this virus in mammalian vertebrates [16]. The first report of *WSLV4* was in the Asian continent, in China, in mosquitoes collected in 2013 without species description by transcriptome sequencing in vertebrates [11]. In mainland Europe, *WSLV4* was detected in *Ae. albopictus* females collected in Catalonia, Spain in 2015 [17]. In 2019, *WSLV4* was detected by metagenomics in populations of *Ae. albopictus* collected in Alexandroupoli, Greece [18], and in the same year, the virus was detected in pools of males in Ticino, Switzerland [19]. More recently, the virus was detected in males and females of *Ae. albopictus* reared in the laboratory from larvae collected in different cities in Germany [20]. *WSLV4* was also detected in adult *Ae. albopictus* collected in 2017 from a residential area in West Valley, California, USA, using metagenomics methodology [21].

In our study, *WSLV4* was detected in three of the four tested pools of female *Ae. albopictus* reared in the laboratory from the collection of eggs in two urban parks in São Paulo. The only negative pool was from females of this species from eggs collected in winter. A study of the *Ae. albopictus* virome captured at different seasons in China showed greater abundance of vertebrate viruses in summer and autumn and greater abundance of vertebrate viruses in winter and spring, with no differences between rural or urban capture sites [22]. The influence of seasons on the presence and abundance of ISVs in *Ae. albopictus* requires further investigation.

Despite the growing number of studies, there is still a gap in knowledge about the influence of ISVs on the ability of mosquitoes to infect and transmit arboviruses. In an experiment using *Ae. albopictus* C6/36 co-infected with *Nhumirim virus* (NHUV), an ISV, and *West Nile virus*, as well as NHUV and *St. Louis virus*, a significant reduction in viral replication of *West Nile virus* and *St. Louis virus* was observed compared to the control group [23]. Similarly, another study investigated the effect of simultaneous infection with NHUV and ZIKV, as well as NHUV and DENV-2, on *Ae. albopictus* C6/36 and observed a significant titer reduction of both arboviruses [24]. The presence of some ISVs in mosquitoes can either reduce or increase the replication of arboviruses in mosquito salivary glands, thereby affecting vector competence [9]. Investigating this relationship may help to understand the difference in vector competence between *Ae. aegypti* and *Ae. albopictus* in nature, for example.

The scarcity of reports on ISVs from *Ae. albopictus* may be related to the lower importance of this species as a vector of pathogens compared to *Ae. aegypti*, as well as the different approaches of metagenomic technology to identify specific viromes of different mosquitoes [16]. *WSLV4* has been associated with *Ae. albopictus*, but it is not clear yet whether it is an ISV exclusive to this species. Virome analysis of 4 pools of *Ae. albopictus* collected in Colombia did not indicate the presence of ISV *WSLV4* [25]. It is necessary to deepen the knowledge of ISVs found in *Ae. albopictus* to evaluate their interaction with invertebrate hosts and other micro-organisms, their complex interaction with different pathogenic arboviruses, and their potential action as biological control agents.

## 5. Conclusions

This is the first detection of *WSLV4* in populations of *Ae. albopictus* from Latin America. Further research on ISVs of *Ae. albopictus* is needed to better understand the interactions between ISVs and pathogenic arboviruses in invertebrate hosts, which may affect arbovirus transmission.

## Figures and Tables

**Table 1:** Summary of mosquito pools used in the study of metagenomics and the genomic results.

ID Sample	32	99	190	332
Species	*Ae. albopictus*	*Ae. Albopictus*	*Ae. albopictus*	*Ae. albopictus*
Number of mosquitoes	10	10	10	10
Gender	Female	Female	Female	Female
Sample collection	Chico Mendes	Lajeado	Chico Mendes	Chico Mendes
Sampling time point	Spring/2018	Summer/2019	Autumn/2019	Winter/19
Number of reads per barcode	328,573	347,619	279,564	183,382
Average quality of reads	9.0	9.0	9.1	9.3
Genome	*Wenzhou sobemo-like virus 4*	*Wenzhou sobemo-like virus 4*	*Wenzhou sobemo-like virus 4*	-
Coverage %	99,96	99,73	100	-
Average depth of coverage x	58,091	91,722	353,239	-
Max depth of coverage x	121	237	940	-
Length of genome (base pairs)	2961	2961	2961	-
Amount of GC (base pairs)	1228	1274	1309	-
Number of reads per genome	383	633	2398	-
N50	2961	2961	2961	-

## Data Availability

FASTA files of genomes the *WSLV4* deposited to NCBI GenBank and are available under the following accession numbers: OP369312, OP369313 and OP369314.
